# Becoming a Guest: On Proximity and Distance in Mental Health Home Treatment

**DOI:** 10.1007/s10606-022-09456-1

**Published:** 2022-12-12

**Authors:** Stefan Hochwarter, Julian Schwarz, Felix Muehlensiepen, Eric Monteiro

**Affiliations:** 1 Department of Computer Science, Norwegian University of Science and Technology, Trondheim, Norway; 2 Center for Health Services Research, Brandenburg Medical School, Rüdersdorf, Germany; 3 University Clinic for Psychiatry and Psychotherapy, Immanuel Clinic Rüdersdorf, Brandenburg Medical School, Rüdersdorf, Germany; 4 Faculty for Health Sciences, Brandenburg Medical School, Neuruppin, Germany

**Keywords:** Distance, Guest, Ethnography, Health care, Home treatment, Mental health, Proximity, Psychiatry

## Abstract

Mental health home treatment is a service where patients with severe mental illnesses are visited by a multiprofessional psychiatric care team at their homes. In Germany, inpatient-equivalent home treatment as a specialized form of home treatment has been offered by hospitals since 2018. In its early stage, the shift of care activities out of the hospital toward the patient’s home opened up a new set of problems and blurred the existing boundaries. This ethnographic study follows two home treatment teams and provides an in-depth description of their work. The findings are presented by three themes from our data analysis: (i) closeness and familiarity; (ii) bridging the distance; and (iii) tensions of proximity and distance. We then discuss the findings with the guiding lens of *Becoming a Guest*, which refers to the ambiguity of proximity and distance. The contribution for computer-supported cooperative work (CSCW) is twofold; on the one hand, we provide a detailed account of mental health home treatment, and on the other hand, we outline a conceptual model that helps to describe and analyze similar cases. We conclude the paper with directions for further research.

## Introduction

The health care activities of almost all disciplines have started to wander again. In the 18th century, the center of care and related activities, including research, increasingly shifted to the clinic (Foucault, 1963). The birth of the clinic entailed not merely the reorganization of health care but, more decisively, a reorganization of medical knowledge and how it was created. After the French Revolution, there was a change of paradigm, away from merely classifying the disease and separating it from the patient; the symptoms were to be experienced with the patient, and the patient became a descriptive part of specifying the disease. ‘This new structure is indicated […] by the minute but decisive change, whereby the question: “What is the matter with you?”, with which the eighteenth-century dialogue between doctor and patient began […], was replaced by that other question: “Where does it hurt?”, in which we recognize the operation of the clinic and the principle of its entire discourse’ (Foucault, 1963, p. xxi). The illness was freed from the strict nosology, which led to freed knowledge. Currently, we see a tendency in the opposite direction: moving care activities back into the patients’ home, but with different means and objectives. At the same time, the structures of the clinic remain stiff and have trouble adapting. Through digitalization, proximity between patients and health care workers is mimicked, thus blurring the temporal, spatial and personal boundaries (Pols and Moser, 2009).

Mental health home treatment (HT), a service where a home treatment team visits the patients in person, sheds light on the blurring of these boundaries. Moving mental health care into a different setting can result in rearrangements of established structures and herewith create new challenges, as Strauss et al. described in their study: ‘When ideologies entailed more radical departures from conventional hospital organizations, however, alterations in professional roles and identities could be quite profound’ (Strauss et al., 1981, p. 157). Our study was guided by the aim of understanding how health care workers carry out these temporal and spatial distributed care activities and what role the various actors/actants play in this endeavor. The general importance in health care of close interaction or proximity between health care workers and patients is especially pronounced in mental care. Mental health is thus a particularly fruitful setting to analyze the technologically mediated proximity, making the relative absence of CSCW attention to mental health unfortunate. Rather than a dichotomous separation of close vs. far, home vs. away and physical vs. digitally mediated, the challenge is rather to ‘understand precisely how the digital can be real’ (Boellstorff, 2016, p. 388). Hence, another objective of this study is to provide an in-depth description of mental health home treatment and its use of digital and physical artifacts.

As the title of this article has already indicated, the overarching theme can be coined by the term *Guest* drawing on its etymological origin, where ‘The guest (ghosti) in Indo-European times was the person with whom one had mutual obligations of hospitality. But he was also the stranger, and the stranger in an uncertain and warring tribal society may well be hostile; the Latin cognate hostis means “enemy”’ (Watkins, 2011, p. xxix). The theme of the guest, in our analysis, is used as a lens to highlight the ambiguous and conflictual meanings of proximity vs. distance in health worker-patient interactions. Our article’s focal points in this two-way relationship are the mental health home treatment teams and how they become guests in the course of their work, i.e., balancing individual needs for proximity and distance in a reiterative mutual process.

## Background and related work

In the past, CSCW has investigated the domain of health care and its various forms of work from different theoretical and empirical perspectives. While the domain of mental health was touched upon in CSCW, it does not have the same body of literature as home care or primary care settings. We start the literature review with the broader perspective of moving care into the homes, followed by previous work on mental health in CSCW, and finish with a theoretical framing and related concepts.

### Moving care into the home

Moving care into the home changes how work in health care is carried out and poses new challenges to its coordination. Fitzpatrick and Ellingsen (2013) noted this in their review of CSCW studies in health care. Although they report mainly from research where information and communication technologies (ICT) were used to provide care at home (telehealth), we see many similarities when health care activities also move physically, i.e., in person but mediated by digitalization, into the homes of the users. In these studies, the patient and the health care worker become the *key participants* and the home becomes the *key site*. The themes addressed included the use of ICT for managing chronic care and the role of the space in the home. The latter might be negotiated, shared, and cooperation with family members is established. CSCW is a design-oriented discipline, and in contrast to institutional settings, a larger body of literature focuses on designing technologies for the home. Fitzpatrick and Ellingsen conclude their review with four suggestions to broaden the research; the second one calls ‘to expand the “work practice” settings in which CSCW research is conducted, to follow the patient trajectory around the multiple settings in which their care is provided and to do this over more extended periods of time’ (Fitzpatrick and Ellingsen, 2013, p. 652). This recommendation aligns with Bratteteig’s and Wagner’s article from the same year. Based on their study of elderly people living independently in their homes, they recommend looking ‘at homecare settings as a whole: at the web of different types of spaces, artifacts, work tasks, people and collaborative patterns that make up these settings’ (Bratteteig and Wagner, 2013, p. 158). We agree with these points, and our study contributes insights into different settings and patient trajectories.

Although the focal point of our study is not primarily the patients’ work at their homes, the numerous studies in CSCW and neighboring fields provide a fitting ground to explore our findings. Connecting to articulation work (Strauss, 1985), illness work, everyday life work, and biographical work (Corbin and Strauss, 1985), Bratteteig and Eide identify other forms of work that play a role when becoming a good homecare practitioner. Relevant for our study is the concept of *information work*. It is described as ‘[a]ll the work that a homecare worker does to gather, process and select information important for the actual work that day (that shift)’ (Bratteteig and Eide, 2017, p. 582). In their case, different information sources are consulted for the work, while the journal system itself is not available on the road.

Care activities at home are seen as more complex than those in a hospital setting. The manifestation of this increased complexity can be observed by the required awareness and articulation work to perform the home care work. A binder, situated at the patient’s home and used by a health care worker, is a central artifact to support coordination. When different care providers are involved, the binder supports awareness across different actors. It facilitates communication both asynchronously and informally, often with the additional help of sticky notes (Petrakou, 2007). The family can play an active part in care and its articulation work. As Christensen and Grönvall (2011) describe, the binder can be designed with the values and attitudes of family members, patients and care workers in mind and by doing so, avoiding possible tensions. Generally, the central role of coordination artifacts in analogous and digital forms was studied from different perspectives within CSCW (Amsha and Lewkowicz, 2016; Bossen et al., 2013).

Moving health care activities into homes further challenges the design of systems that are used for coordinating or documenting care. In a participatory design project, Corry and Larsen identify four challenges for EHR systems in such an environment: personal relations, coordination between primary and secondary health sectors, preventive home measurements, and the distribution of information. They call for a flexible architecture that focuses not only on treatment or care but that can also be used for daily tasks around them (Corry et al., 2006). For an extensive overview of challenges when designing for home care, one can consult the book chapter by Grönvall and Lundberg. They conclude by emphasizing the importance of the environment and changing demographics (Grönvall and Lundberg, 2014).

### CSCW and mental health

Although mental health HT is not a new concept (see Section 3.1), a broader uptake and implementation started only recently. Hence, it is only natural that studies investigating mental health outside the traditional setting focus on aspects other than receiving mental health treatment directly at home. In CSCW, we found that most work focused on three broad themes: social media, peer support/communities, and cultures or cultural influences. Many articles describe design opportunities, often for a specific mental illness or user group.

Studies focusing on mental health and social media are frequently carried out using a quantitative approach, finding different usage patterns of the targeted population. Park et al. found that ‘depressed users have small [social media] networks’ (Park et al., 2015). These findings are in line with a study on user patterns of an online LGBT community, which found that users ‘who are less depressed are more deeply integrated into the social fabric of [the investigated social media platform]’ (Homan et al., 2014). In addition to the predominant theme of social media and depression, although often very vaguely defined, other themes include eating disorders and self-harm. Chancellor et al. presented an algorithm to predict mental illness severity levels based on the use of social media. They collected data on Instagram, marked with the ‘pro-eating disorder tags’ (Chancellor et al., 2016). Feuston and Piper describe an alternative method to look at the social media data beyond purely classifying content. They complete data with interviews and digital ethnography and aptly state that ‘[t]he relationship between mental health and mental illness is thin, porous, and flexible, and not fully observable through behaviors on social media’ (Feuston and Piper, 2018, p. 17). On a conceptional level, Pater and Mynatt aim to set an agenda by introducing a theoretical framework and defining ‘digital self-harm as the online communication and activity that leads to, supports, or exacerbates, non-suicidal yet intentional harm or impairment of an individual’s physical wellbeing’ (Pater and Mynatt, 2017). A good example of a deeper analysis of social media and the role of these platforms for eating disorders can be read in an earlier work of the same authors (Pater et al., 2016). Another perspective on eating disorders and the use of social media is offered by Andalibi et al. They describe how social media and its community can support self-disclosure and ‘engage in social exchange and storytelling about difficult experiences’ (Andalibi et al., 2017).

Peer support in mental health has often been investigated to provide design opportunities for online communities. In the reviewed articles, peers, although not always explicitly referred to as such, are persons who formerly had or are recovering from mental illnesses who take the formal or informal role of a caregiver or adviser. O’Leary et al. identified in their qualitative study how technology can support care in the absence of traditional caregivers and how it could scale to be available for a larger population (O’Leary et al., 2017). By examining design interventions for trauma-related support, Brown and Choi focus on the role of ‘trusted others’ and present six categories for designers to support trusted others (Brown and Choi, 2017). In their interesting, virtual ethnographic study on the largest Chinese online forum for users with mental illnesses, Zhang et al. ask ‘how online support groups are integrated into users’ depression management practices and at the same time create new possibilities for individual empowerment and destigmatization’ (Zhang et al., 2018). Their findings report many benefits, such as patient empowerment and reducing self-stigma.

The paper of Zhang et al. brings us to the next theme: the role of culture. Their first research question investigates the impact of Chinese culture on online support groups in mental health. In their findings, they describe the strong impact culture has on how mental health is perceived by patients, care providers, and society. For instance, the relation to the online support group is intimate, often providing a place for communication without threatening the family’s ‘face’ (Zhang et al., 2018). These findings are in line with the case study of Li et al. on a Chinese online community for people with depression. Additionally, they provide social media design opportunities based on their findings (Li et al., 2016). Using social media as a lens, De Choudhury et al. present the differences in shared social media content in relation to gender and cultural norms (De Choudhury et al., 2017). Another study on social media and depression (Homan et al., 2014) focuses on a youth LGBT community. The study highlights how to address a possibly hard-to-reach population with the help of social media.

There are certainly more articles on mental health in CSCW and even more in neighboring fields such as human-computer interaction (HCI) or information systems (IS). A review of mental health in CSCW and HCI can be found in the article by Ertl et al., which by itself is worth reading and addresses the timely and important topic of refugees’ mental health (Ertl et al., 2020). What we did not find, however, were articles on mental health home treatment. Additionally, most of the articles we found focused on a specific intervention or design, reporting primarily on the patients’ perspective. There was generally a lack of depth on the nature of work in mental health care. Our study aims to fill this gap, at least partially.

### Theoretical framing and related concepts

Coordinating the work of multiprofessional teams, distributed over time and space and addressing the needs of different patients can be an overwhelming and complex task. In describing what role tools play here, Berg decides to conceptualize these ‘reading and writing artifacts’ as artifacts with the capacities to coordinate and accumulate: ‘These activities are closely coupled: the coordination of activities is achieved *through* the accumulation of inscriptions, and vice versa’ (Berg, 1999, p. 387). In doing so, the boundaries between computer-based and paper-based technologies are blurred, and the interrelation of the artifacts and their users move to the foreground. ‘Reading and writing artifacts do not “support” tasks: it is only in the interrelation of staff members’ and artifacts’ activities that the task emerges in the first place’ (Berg, 1999, p. 390f). Nevertheless, focusing on this interrelation does not mean neglecting the intrinsic values built into an artifact. For example, responsibilities and role definitions can be affected depending on the design of an artifact. Artifacts, such as medical records, are not merely a representation of the patient or the case but act as mediators (Latour, 2005) and are intertwined with their surroundings. This affects how the patient is perceived and how care is coordinated (Berg, 1999; Berg and Bowker, 1997; Munkvold et al., 2007).

The guest analogy and the ambiguity of proximity and distance pulled inspiration from a book by Pols (2012). In the second chapter, she analyzes the contrast between warm care and cold technologies. While presenting the case of palliative care that deployed telecare to establish additional contact with the patients, she debunks this opposition bit by bit. It is not the opposing nature of cold technology and warm care that sheds light on why patients have experienced good care with the help of technology. Pols then introduces the notion of fitting and modest aesthetics that helps analyze and explain care at a distance.

## Case and method

### Mental health home treatment

The idea of treating severe mental health crises at home is not new. The origins of HT can be traced back several centuries (Johnson, 2013): For more than 700 years, citizens of the Belgian city of Geel have established a system of family foster care, in which people with mental disorders are taken into their homes and cared for. In the 1930s, psychiatrist Arie Querido in Amsterdam, Netherlands, introduced home visits by a psychiatrist and social worker to prevent admission to a mental asylum. In the 1970s, Leonard Stein and colleagues from Madison, Wisconsin, offered multidisciplinary, team-based, acute psychiatric treatment that approximated today’s forms of HT.

The advantages of avoiding inpatient psychiatric treatment are obvious (Winness et al., 2010, p. 201): the ability to continue with everyday life, to stay in touch with family and friends, to recognize and promote the patient’s resources and to recover in familiar surroundings. The psychiatric ward, on the other hand, is an artificial place where many people with acute crises are concentrated, rigid processes and various rules prevail, and the atmosphere is often restless (Wood and Alsawy, 2016).

Internationally, there is a wide range of outreach treatment concepts that differ in terms of the intensity, complexity, and flexibility of treatment. HT or crisis resolution teams (CRT) are currently the most common concepts. They offer acute psychiatric care at home, usually with at least one contact per day for a period of up to 4 weeks. Sonia Johnson gives an excellent overview of the key organizational characteristics according to the HT/CRT concept (Johnson, 2013). Assertive Community Treatment (ACT) teams, on the other hand, offer longer-term outreach treatment for people with chronic conditions or mental disabilities, with a rather lower contact frequency (Nugter et al., 2016).

Even if evidence shows that HT is feasible, effective, and predominantly preferred by patients and relatives (Murphy et al., 2015), widespread implementation is still pending. In the past two decades, only England and Norway have implemented the CRT model into standard care (Hasselberg et al., 2011; Lloyd-Evans et al., 2018). The Netherlands is now implementing flexible ACT teams at different locations (Nugter et al., 2016).

In Germany, HT was first piloted in Krefeld in 2001 (Bechdolf et al., 2011). Further model projects followed that were based on the ACT concept and specialized in treating psychotic disorders (Lambert et al., 2010). In 2013, new legislation was introduced to promote flexible and integrated treatment (FIT) models, including HT, in at least one hospital in each of the 16 German federal states. After considerable pressure from German psychiatric associations, another second legislation followed in 2018, which allows all 585 psychiatric clinics in Germany to introduce an intensive form of HT. This so-called ‘Inpatient-Equivalent Home Treatment’ (IEHT; German: Stationsäquivalente Behandlung, StäB) is close to the CRT model and is currently implemented by approximately 10% of German clinics. The effectiveness, costs and processes of IEHT are currently being evaluated (Baumgardt et al., 2021).

### Research setting

The case study took place at two departments of psychiatry, psychotherapy, and psychosomatics in two German clinics. Both psychiatric departments offer a variety of services for their users to serve different needs and conditions. One of these services is to provide inpatient-equivalent home treatment (IEHT), better known outside Germany as home treatment for mental health or crisis intervention teams. Patients are referred mainly from other professional services, such as psychiatric outpatient clinics or advisory services. Before treatment begins, HT ensures that patients fulfill the legal regulations for IEHT (Weinmann et al., 2020). In the following, we will briefly introduce the research setting of each department and their implementation of IEHT.

*Department (Dept.) Metropolis* (pseudonym) is part of a public hospital in a metropolitan district of a major German city, serving 280,000 inhabitants and an area of 20 km^2^ (see Table 1). Dept. Metropolis offers a variety of services oriented toward the diverse population they serve. In addition to the ambulance, day clinics (intensive, full-day treatment but without stationary admission), and inpatient services, Dept. Metropolis further provides specialized treatment programs such as services for young adults or people with an immigration background. Regular home treatment services started in April 2018 with one home treatment team, and demand continued to grow. After a year, they established a second home treatment team. The two home treatment teams consisted of 14 health care workers at the time of the study: two senior physicians, two assistant physicians, two psychologists, six nurses, one social worker and one nursing manager.

*Department (Dept.) Lakeside* (pseudonym) is part of a nonprofit hospital in a small municipality located only 35 km away from Dept. Metropolis but belonging to a different state, serving a rural area of 1,613 km^2^ with 240,000 inhabitants (see Table 1). Similar to Dept. Metropolis, it also offers a wide range of services for their users. Due to the large area covered by the department, they have two additional outpatient day clinics situated in relatively populous towns. The department was among the first to offer home treatment as a regular service, starting in May 2018. Dept. Lakeside currently has one home treatment team comprising eight health care workers (at the time of the study in August 2020): one senior physician, one assistant physician, two nurses, one physiotherapist, one social worker and two peer-support workers (PSW). PSWs are people with lived experience of mental distress, often ex-users of psychiatric services that support patients in coping with their everyday lives, mediating between the various stakeholders, and often sharing their own recovery stories (Repper and Carter, 2011).

**Table 1:** Some characteristics of the two catchment areas

	Department Metropolis	Department Lakeside
Location	metropolitan district of a major city	mixed rural and suburban district in eastern Germany
Population	290,000	240,000
Catchment area	20 km^2^	1,613 km^2^
Population density (inhabitants/km^2^)	14,500	149
Average age (years)	38,2	47,7
Immigration background (%)	35.9	3.9

### Research method

The presented research is part of a larger research project designed as a multiple-embedded case study (Yin, 2017) with the aim of investigating health care activities and practices when they move into the homes of patients or service users. While the first case focuses on care at a distance mediated with the help of technologies, the case presented in this paper focuses on the health care practices and work conducted at home by health care workers. We chose an interpretive approach to study this objective. The first author carried out the data collection.

Our study followed an ethnographic approach and data were collected with go-alongs, ‘[a] hybrid between participant observation and interviewing’ (Kusenbach, 2003, p. 463). This allows the researcher to focus on the spatial practices and draw from the experiences of the informants, which seemed appropriate for understanding the work involved in home treatment, as the informants are on the move and the spatial practices were of specific interest. Between the visits, there was time for following up on questions that emerged. The first author followed between one and three informants (three nurses, two senior physicians, one assistant physician, and one peer-support worker), depending on their everyday routines. When following more than one person, it was helpful to overhear their conversations. The first author did not give too specific directions on what to talk about, being more interested in their everyday concerns. In the offices, there was contact with all members of the HT teams. The findings presented in this case are based on two weeks of go-alongs (63 h). Observations took place in March (Dept. Metropolis) and August (Dept. Lakeside) 2020. The research project was registered at the Norwegian Centre for Research Data, and all collected data were encrypted and pseudonymized after transcription. Given the focus on the health care workers, we did not collect any therapeutic details during the patient visits. The patients were informed of the presence of a researcher and could deny access. In some cases, the HT teams decided upfront that a visit was not suitable to be observed.

The first author (FA) approached the field with an open mind and took field notes of various impressions and observations (Emerson et al., 2011). Conversations during go-alongs were not recorded, but jottings were written down. The field notes were transcribed shortly after they were taken, often in the evening of the same day. During transcription, memos were drafted either to follow up on observations or to note early ideas about conceptual perspectives. Over time, the field notes became more focused. In addition, documents and pictures were collected, which were included in the analysis. Initially, the field notes were written in German and subsequently translated into English once they were imported into the computer-assisted qualitative data analysis software MAXQDA.

Data analysis followed a grounded theory approach (Charmaz, 2014). As described above, an inductive approach was taken to see new phenomena of interest in the beginning. Although the FA familiarized himself with home treatment by reading textbooks (Längle et al., 2018; Weinmann et al., 2020) and being in contact with one of the home treatment teams before the fieldwork, it was the first time to see the work of the home treatment teams in practice. Hence, in the beginning, the field notes were broad yet detailed. The FA tried to grasp every detail; any nuance of the specificity of their work, and even notes on colors and smells and their impressions were recorded. Some of the descriptions now feel, after the complete data collection, almost obvious or redundant, but they were perceived relevant at the time. These notes fulfilled a critical purpose: they allowed us to re-experience the field visits and contributed to a vivid data analysis process. This also entailed writing analytic memos throughout the prolonged research. At the second field visit, half a year apart from the first one, the glance was more focused and initial themes and analytic ideas were either confirmed, altered, or dismissed. Early concepts that seemed relevant were written down and further refined over time. Data analysis compromised moving back and forth in a time-intense process between the data (the pictures and documents proved to be very helpful), the field, discussions, and the literature, and refined the themes, which will be presented in Section 4. Early work on this case was also presented at two conferences, and feedback was incorporated (Hochwarter et al., 2019, 2021). To support the data analysis and for easier sharing with the other authors, we have used mainly MAXQDA but also a fanfold paper for visualizing and structuring different themes. Eventually, three main themes were identified: (i) closeness and familiarity, (ii) bridging the distance, and (iii) tensions of proximity and distance. Together, these three themes make up the conceptualization of becoming a guest.

## Findings

### The daily routine

#### Department Metropolis

**Fig. 1:**
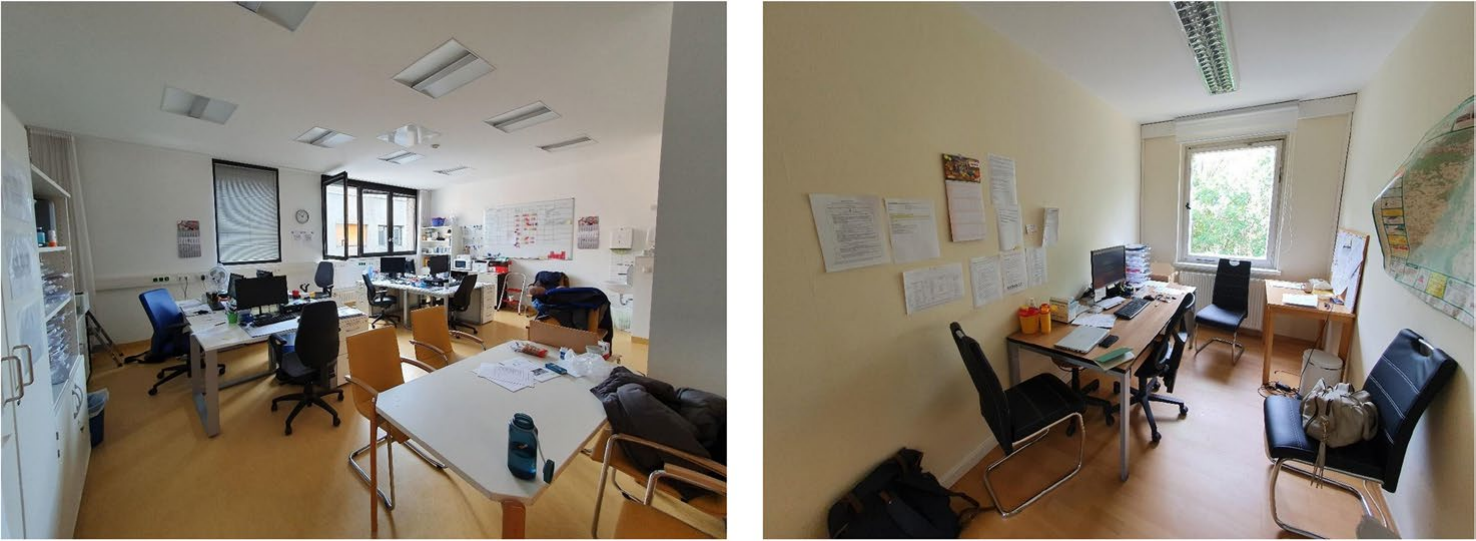
Offices of the HT teams in Dept. Metropolis (left) and Dept. Lakeside (right). These are the larger of the two available offices in each department

Dept. Metropolis hosts two HT teams. They usually start their workday slightly before 8 am, in the larger of their two available offices (see Fig. 1, left). The teams spend the first half of the morning in the office, preparing for the visits and conducting their regular meetings. Their office is configured to support these activities: whiteboards, a table for meetings, the desks groups by teams, and the medication supply stored in a cabinet. During the morning planning meetings, they review each patient, and the visit plan for the next day is decided upon. The team members make notes on paper, in their notebook, calendar or on sticky notes. The information is then written on the team’s whiteboard, consisting of a table with all their active patients. They typically leave their offices between 10 and 11 am. To visit their patients, the HT teams have access to five small cars and two bicycles. While most team members drive a car, one uses his private bicycle and sometimes visits a patient before coming into the office. Before leaving, they arrange the medication for their patients. Usually, they drive without a navigation system; they know the neighborhoods, ongoing construction work and challenging parking situations. They visit the patients alone or in teams of two. The weekly senior physician’s round is one exception, where they visit more patients on a single day with two or three HT workers. When they come back from their visits, typically in the early afternoon, they complete the documentation. Team A has a debriefing meeting, while team B combines this with their morning planning meetings. A nurse from team B describes the different organization with the words, ‘Our team is more independent, but important matters are also communicated when they arise after visits.’

#### Department Lakeside

Dept. Lakeside is home to one HT team that also starts the day at approximately 8 am. The office is in a former apartment building on the edge of the hospital area, just behind the helicopter landing spot. Situated on the top floor, it consists of two office rooms with three desks with one PC in each office. Figure 1 (right) shows the larger of the two offices. Team members work in two groups formed based on their patients’ locations; unlike Dept. Metropolis, they only consist of one HT team. Serving a large area, the two groups are divided to cover a specific geographic area where their patients are located. When preparing, the team members take notes and sometimes check the electronic patient records. There is also a printout of all the patients currently in care. As a result of the morning meeting, today’s visits are documented on a sticky note, including the patients’ names and the planned visiting time. The size of the catchment area (47 × 40 km) and the patients’ scattered location make planning the route a challenging task. On weekends, they work with fewer workers, and two or three team members visit all their patients. Before the HT team leaves the office, they print out a Word document with a patient list. The patients are divided into four tables, depending on which care model the patients are assigned to: StäB (IEHT, standard HT patients, visits every day up to 4 weeks), FIT model (flexible and integrated treatment; a form of integrated care contract that is only accessible to patients belonging to certain health insurance companies; flexible, needs-adapted steering of the frequency of visits), PIA (Psychiatrische Institutsambulanz, psychiatric outpatient clinic; allows approx. one to three visits per patient and quarter), and a waiting list. The HT team has access to two compact cars without any features that could identify them as health care workers. To keep in contact with the other group, they use phone calls or write in a group chat on Signal, an encrypted messaging service. They do not have a debriefing meeting at the end of the day; they usually leave the office early and come back late and try to quickly finish their documentation work.

### Proximity, distance, and tensions

#### Closeness and familiarity

At both departments, the HT teams visit their patients daily for up to eight weeks. They visit the patients mainly at their homes, where they often also meet and include family members. Coming to their homes allows the HT teams to collect impressions they otherwise would not have access to. Furthermore, these frequent visits establish a familiarity between the HT teams and their patients.

The relationship with the patient can be described as intimate. The home treatment team usually sees a patient every day for usually 6 to 8 weeks. Patients are often chronically ill; HT is indicated when their conditions worsen. They are demographically diverse. The youngest patients are 18 years old. The HT team tells me that they get many different impressions from their visits. The family members are often a good assistance and source of information – both actively and passively. (go-along, 24 August 2020)

This proximity leads to an understanding of the patients’ habits. The HT teams know the preferred visiting times, their nuances at their homes, and when to take the shoes off for the visits (I, the first author, made the mistake of bringing shoes with laces). Visiting the patients at home allows for detailed observations.

On the way to the car, assistant physician G says that he observed that the patient was bending down to pick something up, apparently without pain. He has doubts if she truly needs physiotherapy. (go-along, 26 August 2020)

The family members are mostly helpful, providing additional information for the therapy, and sometimes are even actively involved.

We sit outside in the garden in a circle. It’s a sunny and warm day. Senior physician S has the laptop open on his lap. The patient lives temporarily with the family of her daughter after the death of her husband. They have questions regarding the medication that the granddaughter can answer. The granddaughter currently works from home due to COVID-19. She also provides an oral overview of the patient’s history. (go-along, 25 August 2020)

However, sometimes the presence of a family member can also be obstructive. For example, when a family member who is a topic for therapy is eavesdropping.

Similar to the presence of family members, the desired level of proximity is individual. Each patient has a reference therapist, who is the primary contact for the patient and visits the patient regularly. After a new patient is admitted to home treatment, the reference therapist usually initiates regular visits, and together they define an aim for the therapy. During the next week, they get to know their patients well. However, they also try to keep a certain distance in their professional role as therapists.

We found a parking spot close to the first patient without any problems, so we are early for the first appointment. Nurse L tells me that she usually avoids physical contact with patients, but this patient is an exception because she is blind, and she thinks that a hug helps her to create a better image of the person visiting. (go-along, 10 March 2020)

Once a week, the HT teams have a multiprofessional team meeting. Three professional groups, mostly a nurse, a physician and either a psychologist or social worker, discuss all their patients. Even though they bring their handwritten notes and have the EHR system open during these meetings, they often summarize their patients’ progress without consulting any notes:

When senior physician B reports about his patients, he does not need any notes even though his remarks are very detailed. He also knows the administered medication by heart. (go-along, 11 March 2020)

While medication is documented, they sometimes do not need to consult the records when discussing the medication and its dosage. Further details of their visits are shared with other team members, sometimes between meetings and visits, which are not documented. For instance, this can be the patient’s mood, impressions of the visit, or the relationship with their family members. The HT workers also know at what times the patients do not want to be visited.

Another indication of how well they know their patient is the individual setup of the pill dispenser. The HT teams do not use automatic medication dispensers because they do not want to ‘take away the competence of the patient’ (nurse L). They prepare the pill dispenser, stacked with daily medication boxes (morning, noon, evening, bed), which can be withdrawn either from top or bottom:

We visit the last patient on today’s tour. The patient is using HT services for the second time and is currently experiencing a crisis for which she needs HT support. Senior physician W discussed the therapy plan with her. The patient receives the pill dispenser for the week. She withdraws the daily medication box from the dispenser from the bottom, while I have seen most patients withdraw the box from the top. The medication dispenser needs to be arranged accordingly. (go-along, 12 March 2020)

Most patients build a strong trust in HT teams. For instance, a patient was asked by her general physician to deliver a diagnosis once per quarter. The HT team assured the patient that they would not share the diagnosis if she were against it. A similar incidence was observed with another patient. Furthermore, the patients also ask the HT team to support them with tasks not directly related to therapy, such as shopping or writing a recommendation letter for renting an apartment. The supportive relationship to the patients is verbalized, especially during challenging times for the patients, as noted here: ‘You can always call. I’m here to help.’ (assistant physician G) or during another visit:

Nurse K clarifies to the patient that the team is available anytime for him and that he should call on the day of the funeral if he needs help. (go-along, 25 August 2020)

#### Bridging the distance

The proximity and familiarity described in the previous section alternate with a distance between the visits when the HT teams are on the move or in their offices. Although they know their patients well, their work is supported by documents that are regularly consulted and updated, depending on their work routines, patients, and environments.

When visiting the patients, the HT teams of Dept. Metropolis do not have access to their EHR system because ‘we are not online while visiting’. The primary documentation in the EHR system consists of a free-text description of the therapy, some prestructured medical findings (e.g., structured psychopathological finding, a brief systematic description of a person’s mental status at the time of writing), and the performance documentation according to the Operation and Procedure Classification System (OPS). In addition, they also document and prescribe the medication of the patients. On occasion, blood samples, ECG and other clinical tests are taken.

Not all information is recorded in the EHR system. After the HT teams of Dept. Metropolis complete the documentation work, they keep talking about small details experienced with the patients and informally discuss issues at the end of the day.

From 3 pm, the colleagues from the other office also join the office of the nurses, and they informally discuss events from the day and loosely start planning the next day. One patient with financial problems is discussed in detail; they consider supporting him to apply for a special form of early retirement. (go-along, March 10, 2020)

**Fig. 2:**
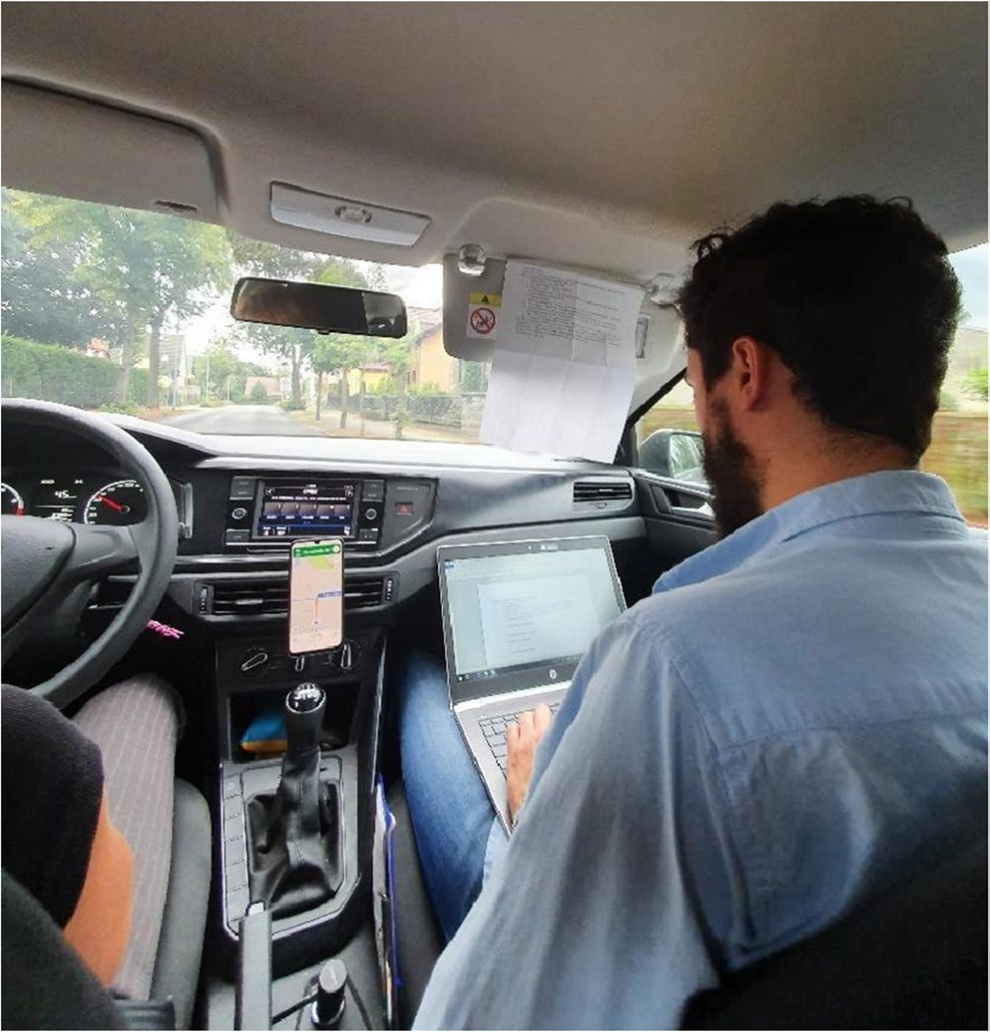
Documentation while driving to a home treatment visit

The HT team in Dept. Lakeside decided to do most documentation during their visits or on the way to/from the visits (see Fig. 2). Hence, they bring a laptop and maintain a Word document for each patient. This document consists of two tables: one for treatment information and one for medication. Treatment information includes the date, a free-text field and the initials of the person documenting. Each entry represents one visit, and the free-text field usually contains the type of visit, the treatment report, psychopathological findings, procedures, and the journey and visit duration. The medication plan at the end of the document is represented by a table with five columns. When prescribing a new medication, a new row at the end of the table is created. Inactive medications are colored in red, and the date and the HT worker’s acronym are noted.

The HT team brings along the laptop to the visits with patients, and during the first part of a visit, the laptop is opened. They often talk to the patient at the dining table or around a coffee table, where they sit across from the patient and rest the laptop on the table. Once they complete the documentation and do not need any further information, they close the laptop but keep talking to the patient. The latter part of the visit is less structured and more open to being led by the patient.

The documentation of therapy is not merely used by the HT team to reconstruct the therapy process or for administrative reasons but also plays an active role during the therapy. However, patients sometimes prefer to keep a distance that the HT team acknowledges. The following observation is from a last regular HT visit.

We arrive at 8:40. The patient welcomes us to the living kitchen in a one-family house. Senior physician S opens the laptop and sits diagonally opposite the patient. S asks the patient if they shall read her therapy transcript together. The patient does not want to, she says: ‘By reading it, it will get worse again.’ S replies: ‘Yes, maybe it is better if you don’t read it.’ (go-along, August 25, 2020)

Furthermore, the documentation also supports the continuity of services outside the HT team’s working hours. Patients, experiencing a crisis, might need to have access to psychiatric services in case of emergencies. While they are treated as inpatient cases, they are situated in their homes, physically remote from the clinical infrastructure. Providing access to the emergency control center can mitigate this distance.

The admission consultation includes the topics of medication, therapy plan, and methods (e.g., exposure therapy). The patient asks who to contact if his condition worsens. He does not want to visit the clinic. Senior physician S explains to him that there are telephone numbers for all team members on the therapy plan. They can be reached during office hours, from 8 am to 4 pm. Outside these hours, the emergency control center can be contacted, and they have a psychologist responsible for the home treatment cases who has access to the patient’s data. (go-along, March 12, 2020)

#### Tensions of proximity and distance

When the HT teams carry out their work, they also encounter friction when balancing the needs of their patients and the resources they have. They face issues of different natures and need to navigate around them to provide services that fit.

Theoretically, the HT team at Dept. Lakeside would have access to the hospital’s EHR system while visiting the patients through connecting to the hospital’s virtual private network (VPN). However, given the inadequate network coverage in the rural area where they operate, this does not work in practice. Furthermore, compared to the Dept. Metropolis, they spend more time driving to the patients; they leave earlier and return later to their offices. Writing documentation before or after the visit does not fit into their schedule. Therefore, for the time being, they implemented a workaround:

Their documentation system is not working on the move because of the region’s inadequate network coverage. The system requires a constant internet connection. In the beginning, they tried to work with it, but this worked poorly. Therefore, they decided to use Microsoft Word (2010) documents instead. For each patient, they create a Word document. In the office, they store USB pen drives with the documents for their patients. In the morning, before they leave to visit the patients, they copy the documents of the patients they will visit on this day to their laptops. That way, they can read the patients’ documentation on the way and document the treatment. At the end of the day or on the next morning, they replace the files on the USB pen drives with their updated versions. Once a week, they transfer the Word documents into the hospital’s system as a report. (go-along, 24 August 2020)

They are aware of the downsides of this workaround and would like to replace this with a ‘proper’ system. Assistant physician G thinks that the documentation is antiquated: ‘I can well imagine that this could be done more efficiently.’ While the Word documents are imported into the EHR system once a week, they are not updated daily, and their content is not searchable. Furthermore, access to these files is only possible within the hospital. This workaround can lead to missing documentation and, in turn, fragments the information they have available. For instance, when an HT worker needs to go home directly after the visits:

We have arranged a meeting with nurse K at a grocery store in a small town to pick up K’s documentation. K will drive home directly after her last visit. At the grocery store, we buy some food and eat at the parking lot. K calls and says that she still has two patients and is late for the meeting. She will send the documentation via e-mail. (go-along, 24 August 2020)

Senior physician S goes through the documentation on the laptop. S: ‘The Word document for patient four is missing.’ Peer-support worker A: ‘Yes, it is still on the laptop. I need to copy this later back and forth.’ (go-along, 25 August 2020)

In addition to the EHR system they use for documenting the therapy, Dept. Lakeside also uses SAP for the reimbursement of their services. Dept. Lakeside also attends to the FIT model patients, who do not need to be visited every day and are treated as outpatient cases. Unlike regular HT patients, they are allowed to visit health care services offered by external providers. While they do not have to be visited every day, during a crisis they are often seen daily. In regard to documentation and administration, the different patient groups follow different regulations. At their weekly team meeting, the HT team at Dept. Lakeside invited a person from the administration to address this issue. The flexible approach and different services provided by the HT team have been a challenge for the administration of the clinic for quite some time now:

It is unclear how the different patient groups of HT should be registered in the system. A person from the administration joined the team meeting and said that she had already said in the beginning that FIT model patients needed to be entered at every visit into the SAP system for billing. That was not done, however. She did not know that the HT team had their own PIA (psychiatric outpatient clinic) patients. They discuss how the HT team should register the patients in SAP. The team only has restricted access to SAP and would like to have full access. The person from the administration says, ‘SAP access is bloody expensive.’ In addition, the HT team also needs to generate a new calendar entry for every patient contact. (go-along, 26 August 2020)

The HT teams of Dept. Metropolis also face challenges using the systems in their clinic for patients located outside the premises. They are offline while driving to or visiting the patients, and the hospital’s EHR system does not work outside the hospital. Therefore, as described earlier, they take notes on paper. At their offices, they use the EHR system that is used for inpatient cases. Each of their two teams has a dedicated ‘virtual room’, where they ‘place’ their patients currently in home treatment.

Furthermore, the handling of a new admission to the service illustrates the interplay of local circumstances and distributed patients. One morning, two suggestions for a new HT admission are proposed. The information about these two patients is printed on paper. The team starts discussing the patients, their medical history, demographic data and where the patients live. One patient lives at the border of the district. The team members complain about the long journey that would entail the admission of this patient. They search the patient’s address; one nurse uses Google maps while another nurse stands up from her desk and walks over to the district map. This district map is pinned on a whiteboard, and the patients’ locations on both teams are marked with colored magnets. A nurse commented, ‘Well, then the patient will be visited at approximately 8 to 9 am during the weekends.’

Home treatment is only one form of treatment offered by the departments. Patients in home treatment might have been inpatient cases before, or after home treatment they might be transferred to the psychiatric outpatient clinic. Hence, when discussing patients in their meetings, they also need access to documentation from different treatment settings provided by the hospital. In these meetings, there is little room for delays.

For the discussion of one patient, they are searching for an overview of the PIA (psychiatric outpatient clinic) appointments in the EHR system. Assistant physician B gives up after slightly less than one minute: ‘No, this is a waste of time. We have to look another time.’ (go-along, 11 March 2020)

Access to information represents not only a challenge with multiple sources, but also a fluent transfer of a patient between home treatment and another treatment setting.

At the end of the meeting, the discharges and admissions are discussed. There is one new admission of a patient; he is currently a stationary patient in the department. This should be a fluent transition from inpatient to HT. They run into some problems about how their EHR system can support this. Assistant physician B proposes to ‘sign the patient up with an offset of one minute.’ Senior physician W said, ‘Then, we cannot invoice it, but ok.’ (go-along, 11 March 2020)

### On becoming a guest

The findings have described HT from various angles, highlighting distinctive characteristics of the work’s nature. Before moving to the discussion, a recapitulation of the findings under the lens of *becoming a guest* is in order.

The individuality of the visits and the demands for the HT teams as guests are reflected already before the visits take place. The offices of the HT teams look strikingly different. The urban catchment area of Dept. Metropolis allows for more planning and documentation work after the visits; hence, their offices hold fully equipped desks and meeting areas to do so. At first glance, the HT offices at the Dept. Lakeside give an impression of a perhaps less organized team. However, this is not the case; their large, rural catchment area requires them to do most of their work in between the visits. Furthermore, the two-way relationship of *becoming a guest* and its characteristics is mirrored by the different forms of treatment that the two departments offer for their patients. Additionally, the multiprofessional teams provide services to the patients based on their specific needs. For example, occupational therapists or social workers are joining the visits to cover all facets and needs.

Our conceptualization deliberately contains a temporal dimension, as the HT teams are *becoming* guests. Closeness, professional or personal, is developed over time. Additionally, between the HT teams and the patients, a closeness is formed over many visits during a period of usually four to six weeks. As guests, they also encounter family members and observe details they would not grasp when the encounter would take place in the hospital. Some patients expressed a strong trust in their visitors. However, the HT teams are more than merely guests. In their professional role as health care workers, their visits serve a purpose. Their gaze observes details that are relevant for the therapy, such as the patient’s living condition and changes from previous visits.

While the visits are time-restricted, the HT teams continue to play the role of guests. The distance in between the visits is often mediated with the help of communication technology and reading and writing artifacts. At Dept. Lakeside, for example, the distributed HT teams coordinate their visits with a messaging service and a shared documentation, which they bring for each visit. The important role of this documentation can be observed when the documentation is not accessible, as described before.

Tensions arise not only when bridging the distance fails but also when there is not the *right* amount of proximity. A (guest) relationship is two-sided, but HT needs to follow certain regulations and rules. Patients expressed their dissatisfaction when the visits did not follow their personal schedule. Although the HT teams try to accommodate this as much as their tight timetable allows, they cannot stay as long as the patient might like. Or on the other hand, the HT regulations do not permit skipping a visit. Hence, while becoming a guest contains both parties in building a relation and power distribution is more equally distributed, the HT teams still have the main responsibility of the visits.

## Discussion

‘A stranger is coming into your apartment with the expectation to hear something new from you. However, from one day to the other, there is not so much change.’ This quote from a discussion with an HT worker fits well with the title of our article: Becoming a Guest. The word guest has its etymological roots in the Proto-Germanic language, and its sources can also be traced to the Latin *hostis*, which can be translated into *strangers* (Watkins, 2011). The transition from being a stranger to becoming a guest requires effort, time, and work to build this reciprocal relationship. However, can this transition ultimately be complete or completed? The findings illustrated that absolute closeness is not the desired aim of mental health home treatment, neither for service users nor for HT workers. Certainly, there is proximity and familiarity, but not all the time and not in all aspects. There are many instances where distance is more beneficial. Hence, this is not a question of proximity *or* distance but rather how to find the right balance of proximity *and* distance, and with what means. In the following, we will discuss this with the help of the previously presented findings.

### Proximity

The transition for HT workers from being a stranger to becoming a guest evolves over the course of HT. Naturally, it takes time to get to know their patients and establish a supportive relationship for their daily visits. The HT teams of both departments visit the patients daily for several weeks, and some patients have also used HT before. As described in Section 4.2.1, this closeness contributes to the HT teams becoming familiar with the patients. During the visits, they pick up different impressions and learn their habits. The therapists recognize small details and nuances, which otherwise might be overlooked. For instance, when a patient, who just complained about back pain, bends down to pick something up. As the therapy is usually carried out at home, family members can become part of the therapy. They are often a source of additional information and even become part of the therapy sessions. Sometimes, however, they may also hinder an open dialogue. At first glance, this obstruction might be perceived as problematic, but understanding the local environments of the patients helps the HT teams to shape a complete understanding of the circumstances the patient is surrounded by.

In this two-sided relationship, the closeness of the patients is often expressed by building strong trust in the HT teams. They share personal details during the therapy, which they do not want other doctors, such as their GP, to have access to. Furthermore, they seek support with practical matters and financial concerns. This closeness, too, can be observed when the patients familiarize themselves with the structure of therapy and acquire competencies, which in turn helps to provide smooth home treatment.

We have observed a closeness that goes beyond mere spatial proximity. There are moments where familiarity is expressed, and this familiarity is built from the beginning of the therapy. The theme of the therapy is defined together; they work together toward a goal. Away from the patient, the HT team remembers small details and even the exact prescription of their medications, often without consulting their notes. Before the end of the day, they talk about their patients informally and provide helpful hints for the next visits. This shows that they maintain mental closeness, even when they are remote.

Specifically, during difficult situations for patients, the HT team makes sure that the patients are aware that this closeness is sustained, even when they are not physically present (‘You can always call. I’m here to help.’). However, closeness is perceived differently. While we have encountered one patient who seeks, due to her blindness, physical contact to better visualize the persons she is interacting with, many patients stated that the visits daily are too much, and early visits are experienced as disturbing. The HT teams try to keep a professional distance while providing a trusting atmosphere. A functioning care relation in an HT setting requires closeness, but not all the time. There is also the need to give room for distance.

### Distance

The treatment of severe mental health conditions in the homes of the patient comes in hand with distance. This distance can sometimes be welcomed, for example when the patients can stay in their homes and do not need to relocate to the clinic. Or when the patient decides not to read through the past therapy transcript together with the HT team. However, a spatial distance does not necessarily result in a relational distance. In overcoming this spatial distance, coordination and accumulation artifacts (Berg, 1999) play an important role. Although the two departments follow different routines for the documentation of treatment, it takes a prominent space within their daily work.

The HT team of Dept. Lakeside must be arranged with a large catchment area and a scattered location of the patients. In their distributed work setting and alternating composition of groups who visit the patients, their work relies, at least in part, on the treatment documentation. Depending on various factors, such as who (which profession) is visiting, the scope of the day’s visit, and the patient’s current state, they prepare for the visit while driving to the patient’s home. The HT team often had visited the patient for several weeks daily and knows their patients well; this helped them to use the limited time for focused discussions. While their in-depth knowledge of the patient forms this picture, they also consult the patient record and the notes from the previous visits. Their work mainly takes place at a distance from the clinic’s digital infrastructure, and hence, they do not have access to the EHR system. Analogous to these reflections in the car, the HT teams in Dept. Metropolis also review their patients before the visits. However, this takes place in the office and not while driving to the patients. With shorter journeys, they can spend more time in their office in the mornings and afternoons, before and after the home visits.

Access to documentation not only mitigates distance for the HT teams, but also allows patients to have contact for emergencies after the service hours of the HT teams. During the daytime, they can reach the HT team and their reference therapist on the phone. One patient expressed concerns about what to do during the night; he needed contact but did not want to come into the clinic due to his condition. Providing access to the documentation for the emergency center helps to provide a continuity of service.

### Finding the right balance

It becomes apparent from the two previous sections that finding the right balance of proximity and distance depends on both the patients and the HT teams, as well as on the environment they act in. Patients have different habits, concerns and needs. The HT teams try to accommodate them as well as they can with the resources at hand. Both departments offer services based on the individual needs of their patients. We have been focusing in this paper on HT, but there are also complementary services that contribute to continuity of care that have other structural and organizational demands. When operating in such an environment, frictions occur.

The importance of having access to the treatment documentation can be observed at Dept. Lakeside, where they implemented a workaround for documentation of the treatment on the move. The two HT groups drove to their patients in parallel and kept in contact using mobile phones. As presented in the findings, they use laptops and a Word file per patient to document the therapy in a somewhat structured format. Additionally, knowledge is spread out over paper notes, sticky notes, patient curves and personnel experiences. The dispersed location of the patients and the changing HT group allocation leads to little space for flexibility throughout the day. Adjusting to changes is difficult, time-consuming and interferes with their work. This causes tensions and issues. In both hospitals, the teams depend on systems and organizational structures designed for work carried out mainly in the hospital and not at a distance. The HT team in Dept. Metropolis handled these issues relatively well, although in their EHR system, they need to assign their patients into ‘virtual rooms’. Usually, EHR systems support the spatial distribution of patients and its arising logistics. Rooms are allocated to patients of the same sex. However, HT patients are not situated in a single room but are distributed in their own homes. Nevertheless, they need to be managed in a single room in the EHR system, which leads to problems with mixed-sex room allocations.

From the patient perspective, the patients themselves also expressed the wish for more flexibility. Some of them wanted to use ambulatory health services but were not allowed to due to the strict regulations that come with HT. Other patients told us they preferred a more flexible service and not being visited every day, and some of the visits could be replaced with phone or video calls. At Dept. Lakeside, they have a different form of therapy (FIT model patients) that allows for greater flexibility. Or, as one of the nurses told me, ‘Model patients are better off, I believe.’ However, providing services that fit the needs of their patients, the HT team at Dept. Lakeside faces increased administrative and organizational workload and conflicts, as we could observe at a meeting with an administrative assistant (Section 4.2.3). Related to this, we can see how Dept. Metropolis struggles with their EHR system when transferring a patient from HT to another service.

### Becoming a guest

We started with the analogy of the etymology of the word *Guest*, and the transition from *being a stranger* to *becoming a guest*. *Stranger*, in our case, represents remoteness, while *Guest* stands for familiarity and proximity. Now we would like to end the discussion with yet another play on words and briefly discuss the oxymoron *distant proximity*. We believe that good mental health home treatment depends on the right balance between proximity and distance, similar to care at a distance, as described by Pols (2012, p.37): ‘Good care requires warmth and coldness, knowledge and empathy, but carers need to mix them in quantities that fit the particular and temporary situation of individual patients.’ The concept of *Guest* that we outlined is the ambiguity about proximity and distance. Good care can be close, while distant at the same time and vice versa.

The right balance of proximity and distance is individual. Patients have different perceptions of closeness and needs. These are not constant; they change over time and vary based on their conditions. For HT teams, finding the right balance of proximity and distance is a recurring task for each patient, and to achieve this, a good understanding of the patients is important. Here, reading and writing artifacts such as patient records play a central role. They can inform the therapy of the patient, providing details from previous visits or allowing reflection on the past together with the patient. Furthermore, they can mitigate the distance when being treated at home by providing the patient’s history to an emergency center that might be consulted by the patient. Good mental health care does not need to be close all the time; many patients asked for fewer visits, and daily visits were sometimes experienced as disturbing and unnecessary. However, the patients appreciate the certainty of having the home treatment teams in reach when they need them.

Furthermore, proximity and distance can be discussed within the guest-host relationship. While the HT teams enter the homes of their patients as guests, they do not do so as understood in the traditional sense. Although they leave the clinic and thereby weaken the prevailing power asymmetry—cf. the keychain worn openly in clinics, see (Längle et al., 2018)—the main responsibility for the visit is still carried by the HT teams and not the patients. Hence, it requires the HT teams to be attentive to the patients and understand when they have overstayed. Otherwise, the visit might be perceived as a burden. When the HT teams succeed in finding this balance together with the patients, the visits are generally perceived as positive and welcomed. However, there are limitations to this endeavor. For instance, the tight schedule the HT teams need to operate in. Furthermore, the closeness, although in a certain situation it might look otherwise, is of a professional nature. Patients understand that the purpose of the visit is to receive therapy, and they decide what to share with their guests. The HT teams, while getting close to the patients, need to maintain professional distance during therapy. Communicating openly these and similar limitations and constraints contributes to positively shaping the space for the visit and the guest-host relationship. In our case, this was done during the initial interview with the patient and the first visits.

## Conclusion

In this article, we have described the work of mental health home treatment based on ethnographic fieldwork at two sites in Germany. We focused on the ambiguity about closeness and remoteness in constructing familiarity with strangers. The (re)configuration of proximity and distance is sustained with the help of technology such as electronic patient records. The frictions we have described—the workarounds, the mix of digital and paper-based notes, being disconnected from the clinic’s infrastructure—are common phenomena within care that moves into the homes (see, for example, Bratteteig and Wagner, 2013). We have drawn from Jeannette Pols’ analogy of cold technology and warm hands, where she lays out the notion of fitting (Pols, 2012). Fitting can also describe a strategy to address the misfits of systems, which is described together with other strategies such as workarounds (Gasser, 1986). For HT teams, balancing proximity and distance involves finding ways to comply with their clinic’s infrastructure and structure, which deviates from their daily work, hence resulting in workarounds (Garfinkel, 1984). In our discussion, we have reflected on how this manifests in proximity and distance.

As we mentioned in the introduction, our aim was to understand how health care workers carry out temporal and spatial distributed care activities in a domain where close interaction between health care workers and patients is in the foreground. The study’s findings suggest that finding the right balance between proximity and distance, often with the help of technology, is a key ingredient to providing care that fits (cf. Pols, 2012). Our main contribution is the conceptualization of this process, which we named *becoming a guest*. Furthermore, we contributed to the body of health care literature in CSCW by introducing mental health home treatment to the domain. To our knowledge, this is the first work on this topic, and we gave an in-depth account of the work in mental health home treatment.

We have chosen to discuss these findings under the term *Guest* because, during the fieldwork, the feeling of *becoming* a guest was omnipresent, not only during the visits but also when observing the work in the departments. We have discussed the findings in light of this inspiration. When we entered the field and started collecting data, our focal point was the HT workers. Over time, while being part of many visits, our perspective widened, and the theme of this paper emerged. Furthermore, we describe care at a distance that is, although coordinated and supported by technology, carried out in person in the homes of the service users. Distance mediated by technology with the help of telecare systems might result in different configurations, and finding the right ingredients for a balance of proximity and distance could imply different steps and considerations.

### Limitations and suggestions for future research

Our study drew mainly from ethnographic data with the HT teams as the center of attention. Hence, our findings naturally shed more light on the HT teams than on the patients. We acknowledge though that mental health home treatment and similar services entail a two-way relationship. Although we collected impressions during the visits, which included both the patients and HT teams, we see the focus on the HT teams as a limitation of our study.

Future research can start where we fell short, i.e., investigating the patient’s perception of mental health home treatment and possibly comparing it with experiences from different forms of mental health care services. Many patients we encountered had previous experiences with inpatient treatment or other forms of therapy offered by the clinics. Moreover, while we encourage more research in mental health care, research on home visits in different domains can also be of interest to further develop our concept.
